# Chewing Stimulation Reduces Appetite Ratings and Attentional Bias toward Visual Food Stimuli in Healthy-Weight Individuals

**DOI:** 10.3389/fpsyg.2018.00099

**Published:** 2018-02-08

**Authors:** Akitsu Ikeda, Jun J. Miyamoto, Nobuo Usui, Masato Taira, Keiji Moriyama

**Affiliations:** ^1^Maxillofacial Orthognathics, Division of Maxillofacial and Neck Reconstruction, Graduate School of Medical and Dental Sciences, Tokyo Medical and Dental University, Tokyo, Japan; ^2^Cognitive Neurobiology, Division of Maxillofacial and Neck Reconstruction, Graduate School of Medical and Dental Sciences, Tokyo Medical and Dental University, Tokyo, Japan

**Keywords:** chewing, attentional bias, visual probe task, eye-tracking, reward circuit, appetite

## Abstract

Based on the theory of incentive sensitization, the exposure to food stimuli sensitizes the brain’s reward circuits and enhances attentional bias toward food. Therefore, reducing attentional bias to food could possibly be beneficial in preventing impulsive eating. The importance of chewing has been increasingly implicated as one of the methods for reducing appetite, however, no studies to investigate the effect of chewing on attentional bias to food. In this study, we investigated whether chewing stimulation (i.e., chewing tasteless gum) reduces attentional bias to food as well as an actual feeding (i.e., ingesting a standardized meal) does. We measured reaction time, gaze direction and gaze duration to assess attentional bias toward food images in pairs of food and non-food images that were presented in a visual probe task (Experiment 1, *n* = 21) and/or eye-tracking task (Experiment 2, *n* = 20). We also measured appetite ratings using visual analog scale. In addition, we conducted a control study in which the same number of participants performed the identical tasks to Experiments 1 and 2, but the participants did not perform sham feeding with gum-chewing/actual feeding between tasks and they took a rest. Two-way ANOVA revealed that after actual feeding, subjective ratings of hunger, preoccupation with food, and desire to eat significantly decreased, whereas fullness significantly increased. Sham feeding showed the same trends, but to a lesser degree. Results of the visual probe task in Experiment 1 showed that both sham feeding and actual feeding reduced reaction time bias significantly. Eye-tracking data showed that both sham and actual feeding resulted in significant reduction in gaze direction bias, indexing initial attentional orientation. Gaze duration bias was unaffected. In both control experiments, one-way ANOVAs showed no significant differences between immediately before and after the resting state for any of the appetite ratings, reaction time bias, gaze direction bias, or gaze duration bias. In conclusion, chewing stimulation reduced subjective appetite and attentional bias to food, particularly initial attentional orientation to food. These findings suggest that chewing stimulation, even without taste, odor, or ingestion, may affect reward circuits and help prevent impulsive eating.

## Introduction

The worldwide prevalence of obesity is increasing, and the condition is a major risk factor for many diseases and premature death (Ng et al., 2014). In recent years, a positive correlation has been found between the speed of eating and body mass index (BMI) (Sasaki et al., 2003; Otsuka et al., 2006; Maruyama et al., 2008; Leong et al., 2011), as well as an association between reduced masticatory function and obesity (Katagiri et al., 2011; Sanchez-Ayala et al., 2013). Therefore, the importance of chewing has been increasingly implicated as one of the methods for preventing overeating.

Food intake is regulated by two complementary drives: the homeostatic and reward pathways (Lutter and Nestler, 2009). The homeostatic pathway controls energy balance with regard to eating after the depletion of energy stores (Lutter and Nestler, 2009). Reward circuits in the mesolimbic dopamine system are driven by environmental cues such as reward, cognitive, and emotional factors (Lutter and Nestler, 2009). In the modern world of plenty, the reward pathway can override the homeostatic pathway by increasing the desire to consume highly palatable foods (Lutter and Nestler, 2009). Therefore, addressing the reward system is considered important.

The visual appearance of food interacts with the brain’s reward circuits and triggers motivated behavior, which plays a significant role in excessive food intake and the resultant obesity (Castellanos et al., 2009). This process can be explained by the concept of “incentive sensitization” proposed by Robinson and Berridge (1993, 2008), in which addictive substances modify the brain’s reward circuits, leading to hypersensitivity to reward-related stimuli. Dopamine plays a key role in influencing communication among the reward circuits, a complex network of cortical and mesolimbic brain structures (Kelley and Berridge, 2002; Castellanos et al., 2009). The release of dopamine in the reward circuits causes feelings of “wanting” (Volkow et al., 2011). “Wanting” evolves into craving, which manifests behaviorally as seeking and consuming (Robinson and Berridge, 1993). This series of rewarding experiences increases salience of the reward-expected stimuli. Attentional bias occurs when the reward-related stimuli acquire the ability to command attention and increase priority of attentional cues associated with motivational goals in cognitive processing (Field and Cox, 2008). It has been proposed that attentional bias to food stimuli may be an outcome of dopamine activation in the reward circuits (Nijs, 2010). A recent study found that a Food Attention Control Training Program reduced attentional bias to food, and also reduced diet failure rates and BMI in the follow-up assessment (Bazzaz et al., 2017). Therefore, further utilization of methods of reducing attentional bias may be beneficial in preventing impulsive eating.

Mastication/chewing could be one factor that increases satiation and reduces food intake (Hetherington and Boyland, 2007; Hetherington and Regan, 2011; Li et al., 2011; Swoboda and Temple, 2013; Miquel-Kergoat et al., 2015). Several studies have reported appetite reduction by sham feeding (i.e., food is chewed but not eaten) (Wijlens et al., 2012) and gum-chewing (also a type of sham feeding) (Schuster et al., 2006; Xu et al., 2015; Lee et al., 2016). Such chewing stimulation includes motor activities of the jaws, saliva secretion, and intra- and extra-oral somatosensory stimulation. In a systematic review of the effects of chewing on appetite using the meta-analysis approach, some studies reported that chewing stimulation could reduce subjective appetite and influence the metabolic appetite regulation system, including secretion of gut hormones (Li et al., 2011; Zhu et al., 2013; Miquel-Kergoat et al., 2015; Xu et al., 2015). Recent evidence also supports the idea that metabolic signals exert effects on the motivation to obtain food through the regulation of mesolimbic dopamine signaling, i.e., reward circuits (Lutter and Nestler, 2009; Skibicka, 2013). Therefore, chewing stimulation presumably affects not only metabolic signals but also reward circuits, and in turn reduces attentional bias to food. However, to our knowledge there are no studies that investigate the effect of chewing on attentional bias to food.

Therefore, the aim of the present study is to verify whether chewing stimulation reduces attentional bias to food, which reflects the reward pathway, in normal-weight participants. In Experiment 1, as a preliminary investigation of whether chewing affects attentional bias to food, we measured RT in a VPT, which is an index of attentional bias. In Experiment 2, we used ET as the method for accurately assessing changes in gaze direction (index of initial visual attention bias) and gaze duration (index of maintained visual attention bias). Furthermore, we investigated whether chewing stimulation by itself, even in the absence of taste, odor or ingestion showed similar effects to the actual feeding condition on subjective appetite and attentional bias to food. In addition, we conducted a control study to determine the baseline condition in which participants did not perform sham feeding with gum-chewing/actual feeding between tasks.

## Materials and Methods

### Participants

Participants in Experiment 1 (11 males and 10 females, mean age = 25.9 ± 4.7) completed the VPT, and participants in Experiment 2 (16 males and 12 females, mean age = 25.7 ± 3.1) were subjected to ET. Eight participants in Experiment 2 were excluded because they had unusable eye-tracking data, or their proportion of acquired samples was less than 60% (Graham et al., 2011). The proportion of acquired samples of the participants in Experiment 2 is shown in Supplementary Table S1. The final sample in Experiment 2 comprised 20 participants (13 males and 7 females, mean age = 25.8 ± 2.9). Differences in the characteristics of participants between Experiments 1 and 2 were examined using a Mann–Whitney *U* test (α = 0.05). No significant differences were found in terms of age, weight, or BMI.

The control groups of Experiment 1 (12 males and 9 females, mean age = 29.0 ± 3.6) and Experiment 2 (11 males and 12 females, mean age = 29.5 ± 5.1) were subjected to identical tasks to Experiments 1 and 2, but the participants did not perform sham feeding with gum-chewing/actual feeding between tasks and they took a rest. Three participants in the Experiment 2 control group were excluded because of unusable eye-tracking data, and the final sample in the Experiment 2 control group comprised 20 participants (10 males and 10 females, mean age = 29.9 ± 5.3). No significant differences were found in terms of age, weight, or BMI between Experiment 1 and Experiment 2 control group.

All participants fulfilled the following criteria, as screened by a questionnaire: (i) no history of metabolic, neurological, or psychiatric diseases, nor any eating disorders; (ii) no use of any medication; (iii) no current or recent efforts to lose weight; (iv) not pregnant; and (v) BMI < 25 kg/m^2^. The participants received a description of the experimental procedure, but were not informed about the purpose of this study. All procedures in this study complied with the Code of Ethics of the World Medical Association (Declaration of Helsinki) and the standards established by the Institutional Ethical Review Board of Tokyo Medical and Dental University (approval #D2014-063). All participants provided their written informed consent prior to inclusion. A power analysis was conducted by G^∗^Power Version 3.1 (Heinrich-Heine-Universität, Düsseldorf, Germany) to justify the sample size with effect size *f* = 0.4 (η^2^ = 0.14, referring to the previous study by Castellanos et al., 2009), α = 0.05, and 1 - β = 0.8.

Further details regarding trials are provided in the Supplementary Information.

### Study Design

The overview of the study is shown in Supplementary Figure S1. In Experiment 1, a VPT and subjective ratings of appetite, using visual analog scales (VASs), were conducted. In Experiment 2, ET (with VPT) and VAS ratings were conducted. Participants in the two experiments attended on two separate days: one was a sham-feeding day with a gum-chewing session, and the other was an actual feeding session. The order of the sessions was counterbalanced across the participants. In both sessions, the relevant measures were performed twice: once before and once after either chewing gum (sham feeding condition) or ingesting a standardized meal (actual feeding condition). In addition, subjective appetite was rated four times in each session using VAS: before the first VPT or ET (T1); before sham feeding or actual feeding (T2); after sham feeding or actual feeding (T3); and after the second VPT or ET (T4). In both experiments, the participants started chewing gum or ingesting a meal immediately after finishing VAS of T2. Five minutes after T2, participants started completing VAS of T3.

In the sham feeding session, participants were instructed to chew an odorless and tasteless gum base (LOTTE Co., Ltd., Tokyo, Japan) for 5 min to remove any effect other than chewing stimulation. Each participant chewed gum in time with a metronome set at 100 beats/min. In the actual feeding session, participants ingested a fruit-flavored energy supplement (CalorieMate^®^, Otsuka Pharmaceutical Co., Ltd., Tokyo, Japan; 200 kcal/40 g) until they felt satiated. Participants were instructed in advance to fast for at least 10 h before the experiment to create conditions of hunger. They undertook the experiment between 8:00 a.m. and 11:30 a.m., and the average fasting time was 12.4 ± 1.6 h.

### Image Selection

We conducted preliminary experiments to select the stimulus images. In the pre-experiment, 60 images, portraying various types of appetizing high-calorie foods (e.g., pizza, hamburgers, and steaks), were selected from the Internet. These images were rated by a group of 20 individuals (9 males and 11 females, mean age = 23.8 ± 1.54) for valence (level of pleasantness or unpleasantness), arousal (from calm to excited), and the subjective feeling of appetite. Individuals from the pre-experiment were not included in the main study. The ratings of valence and arousal were made with the Self-Assessment Manikin system (Lang et al., 2008), and the ratings of the subjective feeling of appetite were made on a 5-point scale. For valence, 1 represented “unpleasant” and 5 represented “extremely pleasant.” For arousal, 1 represented “calm” and 5 represented “excited.” Concerning the ratings of appetite, 1 denoted “This food is not appetizing for me; I would not like to eat this.” and 5 denoted “This food is very appetizing; I would really like to eat this food.” Sixty non-food images (e.g., tools and furniture) were selected for the target trials from the International Affective Picture System (IAPS) (Lang et al., 2008), which is designed to provide a standardized set of pictures with ratings on the dimensions of valence and arousal. In addition, 80 images of non-food contents (e.g., street scenes and nature scenes) were selected for filler trials from the IAPS, and were interspersed with target trials to vary the task and reduce monotony.

Regarding the target trials, the 60 food images were divided into four sets. Similarly, the 60 non-food images for the target trials and 80 non-food images for the filler trials were also divided into four sets. The mean ratings of the sets for each stimulus type (food, non-food, filler) on each rating (valence, arousal, appetite) are shown in Supplementary Table S2. Within each set, 15 pairs of food and non-food images were matched as closely as possible with regard to color, complexity, and size. For the filler trials, 10 image pairs were made in the same way. Four sets of visual stimuli were allocated to each of four VPT or ET sessions, and stimulus sets were counterbalanced across participants.

### Tasks

#### Appetite Ratings

The ratings of the subjective feeling of appetite were made using four 100-mm VASs (Zhu et al., 2013). Participants were presented with a series of questions: (i) How hungry do you feel right now?; (ii) How full do you feel right now?; (iii) How preoccupied with food are you right now?; and (iv) “How strong is your desire to eat right now?” The VASs were anchored by diametrically opposed statements at each end (e.g., not hungry at all; as hungry as I have ever felt). Participants were instructed to draw a vertical line on the scale at the position that reflected the current strength of their feeling of appetite.

#### Visual Probe Task

In Experiment 1, following published studies using the VPT, we used an image presentation duration of 500 ms (Mogg et al., 1998; Field and Cox, 2008; Nijs et al., 2010).

**FIGURE 1:**
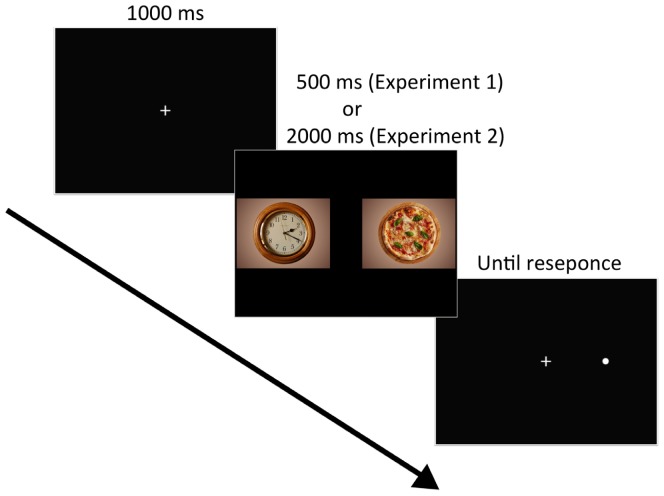
Example of trial events. Each trial displayed the fixation cross (1,000 ms) followed by the paired images (500 ms or 2,000 ms) and the probe, which remained until a response occurred. Image source: https://pixabay.com/en/clock-wall-clock-clock-face-pointer-2634551/ (left image in the middle), https://pixabay.com/en/pizza-food-italy-3000285/ (right image in the middle). License: CC Public Domain.

The VPT included 100 experimental trials: 60 target trials and 40 filler trials. Each trial commenced with a central fixation cross for 1,000 ms, followed by a pair of images for 500 ms (**Figure 1**). Each food–non-food image pair was presented four times, with each food image appearing equally often on the left side and the right side. Immediately after the image pair disappeared, a dot probe appeared at the location of one of the images. The position of the probe was equally distributed between food and non-food images and the left and right sides of the screen. The dot probe remained until the participant responded. Participants were instructed to look at the fixation cross at the start of each trial and to respond to the probe by pressing either the right or left button on a response box as quickly and accurately as possible. In general, RT to the probe provides an index of attention to the image; for example, if participants are attending to the food image, their RT should be faster to a probe that replaces the food image than to one that replaces the non-food image (Castellanos et al., 2009). The order of the images was randomized for each participant.

Reaction time bias scores were calculated by subtracting the mean RTs of the food-relevant trials in which probe replaced food image (RT food) from the mean RTs of the non-food-relevant trials in which probe replaced non-food image (RT non-food). Positive values signified attentional bias toward the food images, and negative values signified attentional bias toward the non-food images (Mogg et al., 1998; Castellanos et al., 2009).

#### Eye-Tracking

Eye-tracking was conducted including the VPT for participants of Experiment 2 to measure aspects of attention more accurately. Following published studies using ET, we presented the images for 2,000 ms (Castellanos et al., 2009; Nijs et al., 2010; Doolan et al., 2014). The data were recorded with a Tobii X60 apparatus (Tobii Technology, Inc., Pittsburgh, PA, United States) comprising a table-mounted camera positioned below the monitor and a system that utilized near-infrared pupil-center/corneal-reflection ET (Weigle and Banks, 2008). The ET data were sampled every 16.7 ms (60 Hz). Prior to beginning ET, participants’ eye movements were calibrated using a 9-point calibration procedure (Doolan et al., 2014).

We calculated gaze direction bias scores and gaze duration bias scores (Castellanos et al., 2009; Nijs et al., 2010; Doolan et al., 2014). Gaze direction bias is an index of initial attentional orientation to a food image (Castellanos et al., 2009). It was calculated as the proportion of the number of trials in which the first fixation was directed to a food image out of the number of all trials in which the fixation was directed toward either the food or non-food image. Gaze duration bias is an index of maintained attention to food (Castellanos et al., 2009). It was calculated as the average proportion of gaze duration toward a food image out of the average total gaze duration toward all images (food and non-food). The fixations that occurred outside of the image pairs (e.g., on a blank screen or not on the screen) were excluded from the data analysis.

#### Control Study

Additionally, we conducted a control study to determine the baseline condition in which the same number of participants performed the identical tasks to Experiments 1 and 2, but the participants did not perform sham feeding with gum-chewing/actual feeding between tasks and they took a rest. The data for the control study was collected with different participants to Experiments 1 and 2.

### Data Analysis

The subjective appetite ratings (VAS scores) were standardized as a *z*-score for each participant to allow for variations among participants. The appetite ratings were focused mainly on changes in T2 and T3, immediately before and after the intervention. However, the appetite ratings of T1 (before the first VPT or ET) and T4 (after the second VPT or ET) were also evaluated because visual food stimuli during VPT or ET might have changed the appetite ratings. For statistical analysis, 4 (time: T1–T4) × 2 (condition: sham feeding vs. actual feeding) two-way repeated measures ANOVAs and *post hoc* Ryan’s tests were conducted to compare participants’ appetite levels.

For the RTs on the target trials in the VPTs, incorrect responses, RTs of less than 200 ms or greater than 1,500 ms, and RTs greater than 2 SDs above each participant’s mean were excluded from analysis (Mogg et al., 1998; Castellanos et al., 2009; Nijs et al., 2010). For statistical analysis of VPT, 2 (time: before vs. after sham feeding/actual feeding) × 2 (condition: sham feeding vs. actual feeding) two-way repeated measure ANOVA were used.

With regard to ET, gaze fixations from the target trials were analyzed using the Tobii Studio software (Tobii Technology, Inc., Pittsburgh, PA, United States). Gaze fixations were defined as saccades that remained within either the food image or the non-food image for ≥100 ms and that were initiated at least 100 ms after image onset. Because eye movements occurring within 100 ms after image onset were considered to reflect anticipatory eye movement, we did not include these data in the analysis (Castellanos et al., 2009; Doolan et al., 2014). For statistical analysis of ET, 2 (time: before vs. after sham feeding/actual feeding) × 2 (condition: sham feeding vs. actual feeding) two-way repeated measure ANOVA were used.

For the control study, each VAS scores or attentional bias scores from VPT and ET were compared among time points using one-way repeated ANOVA followed by Ryan’s tests.

All statistical analyses were conducted with a significance level of 0.05. Effect size was calculated using the following formula:

$$\eta^{2}={df}_{A}\times F_{A}/\left({df}_{A}\times F_{A}+{df}_{E}\right)$$

where *df_A_* and *df_E_* are the degrees of freedom for between A groups and errors, respectively, the *F_A_* value being the one for the A effect (Cohen, 1973; Suzukawa and Toyoda, 2012).

$$r=\sqrt{t^{2}/\left(t^{2}+df\right)}$$

where *t* and *df* represent *t*-test value and degrees of freedom, respectively (Schulze, 2004).

An additional Bayesian analysis was conducted using JASP software (The JASP Team, Amsterdam, 2017; Version 0.8.5). We ran within-subject Bayesian paired *t*-tests to compare between T2 vs. T3 for appetite ratings, and to compare between before vs. after sham feeding, actual feeding, and resting conditions for RT bias scores, as well as gaze direction and gaze duration bias scores. Moreover, we calculated the difference between before and after task scores for the RT bias (Experiments 1 and 2), gaze direction bias, and gaze duration bias, and ran within-subject Bayesian paired *t*-tests to compare the sham feeding and actual feeding conditions. The data were not analyzed to compare the sham/actual feeding vs. control conditions because they were collected at different times with different participants, and these studies were not powered to examine between-subjects differences. To examine relationships between appetite ratings (the difference between T2 and T3 in ratings of hunger, fullness, preoccupation with food, and desire to eat) and attentional bias (the difference between before and after in RT bias, gaze direction bias, and gaze duration bias), Pearson correlations were computed. The pearson’s correlation coefficient between subjective appetite and attentional bias scores are shown in Supplementary Table S3 (see Supporting Information).

## Results

### Experiment 1

#### Appetite Ratings

**FIGURE 2:**
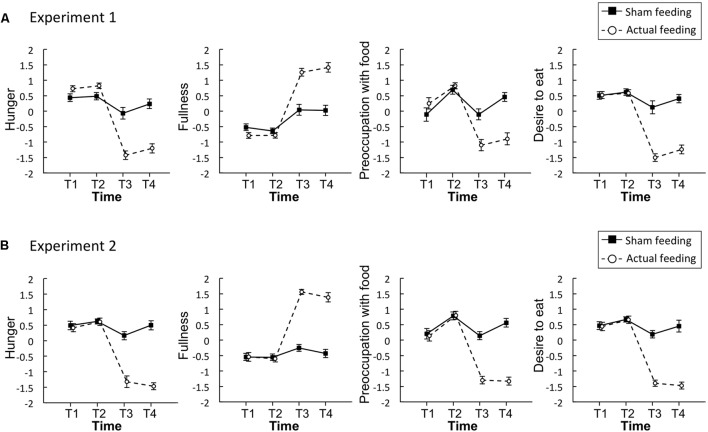
Time series of the standardized visual analog scales (VASs) for appetite ratings of hunger, fullness, preoccupation with food, and desire to eat in Experiment 1 **(A)** and 2 **(B)**. Solid lines represent sham feeding with gum-chewing sessions, and dashed lines represent actual feeding sessions. Error bars represent the standard error of the mean. T1, before the first visual probe tasks (VPT) or eye-tracking (ET); T2, before sham feeding or actual feeding; T3, after sham feeding or actual feeding; and T4, after the second VPT or ET.

**Figure 2A** illustrates the mean standardized scores of subjective appetite from Experiment 1. In all appetite ratings, significant main effects of time and condition, and a significant interaction were observed (*p* < 0.001, **Table 1**).

**Table 1:** Results of two-way ANOVA for effects of time and condition on appetite ratings.

		Main effect of time points			Main effect of conditions			Interaction		
				
		*F*	*df*	η^2^	*F*	*df*	η^2^	*F*	*df*	η^2^
										
			(*df1, df2*)			(*df1, df2*)			(*df1, df2*)
Experiment 1	Hunger	2.33	3, 60	0.84	0.74	1, 20	0.35	1.12	3, 60	0.55
	Fullness	2.71	3, 60	0.88	0.83	1, 20	0.41	1.01	3, 60	0.50
	Preoccupation with food	0.98	3, 60	0.49	0.67	1, 20	0.31	0.84	3, 60	0.41
	Desire to eat	1.58	3, 60	0.71	1.49	1, 20	0.69	1.07	3, 60	0.53
Experiment 2	Hunger	1.70	3, 57	0.74	1.28	1, 19	0.62	1.28	3, 57	0.62
	Fullness	1.70	3, 57	0.74	1.52	1, 19	0.70	1.74	3, 57	0.75
	Preoccupation with food	1.66	3, 57	0.73	1.06	1, 19	0.53	1.32	3, 57	0.64
	Desire to eat	1.99	3, 57	0.80	1.35	1, 19	0.65	1.34	3, 57	0.64


*η^2^, effect size; *F*, *F*-value; *df1* and *df2*, degree of freedom.*

In the sham feeding condition, significant simple main effects of time were observed for hunger [*F*(3,120) = 4.3, *p* = 0.006, η^2^ = 0.10], fullness [*F*(3,120) = 9.4, *p* < 0.001, η^2^ = 0.19], and preoccupation with food [*F*(3,120) = 5.5, *p* = 0.001, η^2^ = 0.12]. There was a marginal main effect on desire to eat [*F*(3,120) = 2.4, *p* = 0.067, η^2^ = 0.06]. A *post hoc* Ryan’s test revealed significant decreases between T2 and T3 in hunger [*t*(120) = 3.2, *p* = 0.002, *r* = 0.28] and preoccupation with food [*t*(120) = 3.3, *p* = 0.001, *r* = 0.29], and a significant increase in fullness [*t*(120) = 4.1, *p* < 0.001, *r* = 0.35] (**Table 2**).

**Table 2:** Mean standardized appetite ratings (standard errors) as assessed at four points in time during the experiment.

			Time points				
				
			T1	T2	T3	T4	Significant difference
							
			Mean ± SE	Mean ± SE	Mean ± SE	Mean ± SE	
Experiment 1	Hunger	Sham feeding	0.43 ± 0.12	0.48 ± 0.12	-0.07 ± 0.19	0.24 ± 0.15	b,d
		Feeding	0.73 ± 0.10	0.82 ± 0.08	-1.43 ± 0.14	-1.20 ± 0.15	b,c,d,e
	Fullness	Sham feeding	-0.52 ± 0.10	-0.64 ± 0.08	0.04 ± 0.17	0.02 ± 0.16	b,c,d,e
		Feeding	-0.79 ± 0.09	-0.78 ± 0.08	1.26 ± 0.12	1.41 ± 0.15	b,c,d,e
	Preoccupation	Sham feeding	-0.11 ± 0.21	0.69 ± 0.14	-0.11 ± 0.18	0.46 ± 0.14	a,d
	with food	Feeding	0.25 ± 0.18	0.82 ± 0.10	-1.10 ± 0.18	-0.90 ± 0.19	a,b,c,d,e
	Desire to eat	Sham feeding	0.51 ± 0.12	0.61 ± 0.10	0.12 ± 0.20	0.40 ± 0.13	
		Feeding	0.50 ± 0.11	0.59 ± 0.10	-1.50 ± 0.12	-1.24 ± 0.14	b,c,d,e
Experiment 2	Hunger	Sham feeding	0.49 ± 0.14	0.62 ± 0.08	0.16 ± 0.13	0.49 ± 0.15	
		Feeding	0.42 ± 0.13	0.61 ± 0.12	-1.32 ± 0.18	-1.47 ± 0.11	b,c,d,e
	Fullness	Sham feeding	-0.55 ± 0.11	-0.56 ± 0.10	-0.25 ± 0.11	-0.43 ± 0.13	
		Feeding	-0.54 ± 0.14	-0.60 ± 0.10	1.56 ± 0.08	1.38 ± 0.15	b,c,d,e
	Preoccupation	Sham feeding	0.20 ± 0.16	0.78 ± 0.12	0.14 ± 0.13	0.56 ± 0.14	a,d
	with food	Feeding	0.14 ± 0.16	0.80 ± 0.13	-1.30 ± 0.12	-1.33 ± 0.12	a,b,c,d,e
	Desire to eat	Sham feeding	0.47 ± 0.12	0.67 ± 0.08	0.19 ± 0.12	0.45 ± 0.19	d
		Feeding	0.44 ± 0.14	0.66 ± 0.12	-1.40 ± 0.10	-1.47 ± 0.11	b,c,d,e


*Significant difference, a: T1 vs. T2, b: T1 vs. T3, c: T1 vs. T4, d: T2 vs. T3, e: T2 vs. T4, f: T3 vs. T4; SE, standard errors.*

In the actual feeding condition, significant simple main effects of time were observed for hunger [*F*(3,120) = 100.4, *p* < 0.001, η^2^ = 0.72], fullness [*F*(3,120) = 110.4, *p* < 0.001, η^2^ = 0.73], preoccupation with food [*F*(3,120) = 28.6, *p* < 0.001, η^2^ = 0.42], and desire to eat [*F*(3,120) = 68.4, *p* < 0.001, η^2^ = 0.63]. *Post hoc* Ryan’s tests revealed significant decreases between T2 and T3 in hunger, preoccupation with food, and desire to eat, and a significant increase in fullness (*p* < 0.001 for all, **Table 2**).

The Bayes factors of appetite ratings for the comparisons between T2 vs. T3 were presented in **Table 3**. In the sham feeding condition, the Bayes factors of all appetite ratings support the alternative hypothesis; fullness and preoccupation with food support strong evidence, hunger supports moderate evidence, and desire to eat supports anecdotal evidence for alternative hypothesis. In the actual feeding condition, the Bayes factors of all appetite ratings support extreme evidence for alternative hypothesis (Schönbrodt and Wagenmakers, 2017). In Experiments 2, 8 participants were excluded due to a lack of sufficient ET data. We removed these data from all the tasks to match the sample numbers among participants. When the data of the 8 excluded participants were included in the visual analog scales (VASs) and VPTs, the results were reported in supporting information (Supplementary Figure S2).

**Table 3:** The Bayes factors of appetite ratings for the comparisons between T2 vs. T3 under the conditions of hunger, fullness, preoccupation with food, and desire to eat.

	Sham feeding	Actual feeding	Resting (no feeding)
**Experiment 1**			
Hunger	3.12	3.92 × 10^10^	0.37
Fullness	12.89	4.86 × 10^8^	0.24
Preoccupation with food	21.70	7.35 × 10^5^	0.25
Desire to eat	1.19	5.71 × 10^7^	0.23
**Experiment 2**			
Hunger	9.64	3.77 × 10^5^	0.34
Fullness	4.56	1.01 × 10^11^	1.50
Preoccupation with food	40.77	4.93 × 10^8^	0.26
Desire to eat	52.40	5.99 × 10^8^	0.27


#### RT Bias Scores

**FIGURE 3:**
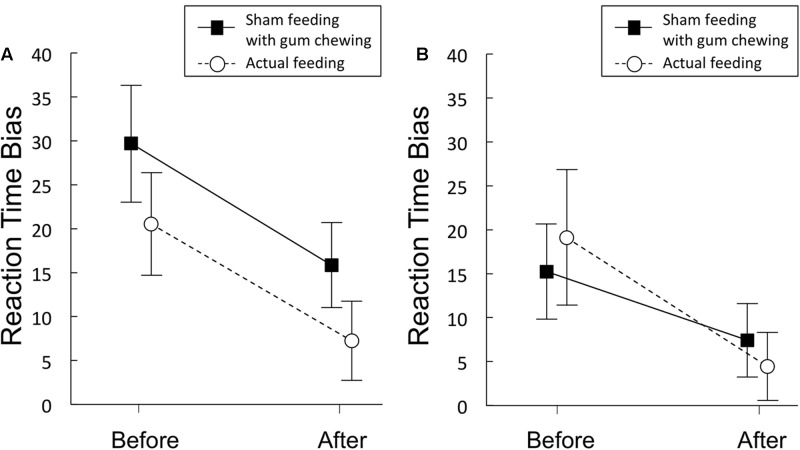
Reaction time bias scores (in ms) obtained from the visual probe tasks in Experiment 1 **(A)** and 2 **(B)**. Solid lines represent sham feeding with gum-chewing sessions, and dashed lines represent actual feeding sessions. The food and non-food images were presented for 500 ms **(A)** or 2,000 ms **(B)**. Error bars represent the standard error of the mean.

**Figure 3A** and **Table 4** shows the magnitude of the RT bias in 500 ms trials to food images. For RT bias, there was a significant main effect of time [*F*(1,20) = 7.3, *p* = 0.014, η^2^ = 0.27] but no significant main effect of condition [*F*(1,20) = 3.2, *p* = 0.09, η^2^ = 0.14], nor any interaction between the two factors [*F*(1,20) = 0.01, *p* = 0.93, η^2^ = 0.0003], indicating that sham feeding with gum-chewing reduced RT bias scores as well as the actual feeding condition did.

**Table 4:** Mean scores on attention-related measures before and after sham feeding or actual feeding.

	Sham feeding session		Feeding session	
		
	Before	After	Before	After
				
	Mean ± SE	Mean ± SE	Mean ± SE	Mean ± SE
**Experiment 1 (Stimulus duration = 500 ms)**				
RT food	325.60 ± 11.62	308.79 ± 9.51	318.89 ± 14.81	305.00 ± 11.76
RT non-food	355.30 ± 14.86	324.64 ± 11.27	339.42 ± 18.18	312.23 ± 11.74
RT bias	29.71 ± 6.58	15.84 ± 5.83	20.54 ± 4.81	7.23 ± 4.50
**Experiment 2 (Stimulus duration = 2,000 ms)**				
RT food	354.60 ± 11.91	353.96 ± 5.83	349.41 ± 11.99	351.65 ± 12.21
RT non-food	369.83 ± 14.13	361.38 ± 14.22	368.5 ± 15.67	356.09 ± 12.32
RT bias	15.23 ± 5.35	7.42 ± 4.19	19.09 ± 7.69	4.44 ± 3.86
Gaze direction food	37.65 ± 1.24	33.90 ± 1.52	34.9 ± 1.79	32.35 ± 1.24
Gaze direction non-food	21.55 ± 1.25	22.80 ± 1.53	24.10 ± 1.93	25.95 ± 1.63
Gaze direction bias	0.64 ± 0.02	0.60 ± 0.02	0.61 ± 0.03	0.56 ± 0.02
Gaze duration food	757.28 ± 54.31	728.71 ± 57.28	797.38 ± 50.49	728.60 ± 44.84
Gaze duration non-food	423.57 ± 29.50	427.82 ± 39.54	428.48 ± 36.02	491.84 ± 35.73
Gaze duration bias	0.64 ± 0.02	0.62 ± 0.02	0.65 ± 0.02	0.60 ± 0.02


*RT food/non-food: mean reaction time (in ms) in food-relevant/non-food-relevant trials in visual probe task. RT bias: difference reaction time (in ms) in food-related and non-food-related trials. Gaze direction food/non-food: the average number of trials when the first fixation was directed to the food image across all trials. Gaze direction bias: the proportion of the number of trials in which the first fixation was directed to a food image out of the number of all trials in which the fixation was directed toward either the food or non-food image. Gaze duration food/non-food: the average time spent gazing at food/non-food (in ms) per trial. Gaze duration bias: the average proportion of gaze duration toward a food image out of the average total gaze duration toward all images (food and non-food).*

The Bayes factors of RT bias in 500 ms trials for the comparisons between before vs. after sham/actual feeding conditions were presented in **Table 5**. The Bayes factors of sham/actual feeding conditions support anecdotal evidence for alternative hypothesis (Schönbrodt and Wagenmakers, 2017).

**Table 5:** The Bayes factors of attentional bias scores for the comparisons between before and after sham feeding, actual feeding, and resting conditions.

	Sham feeding	Actual feeding	Resting (no feeding)
**Experiment 1 (Stimulus duration = 500 ms)**			
RT bias	1.72	1.84	0.25
**Experiment 2 (Stimulus duration = 2,000 ms)**			
RT bias	0.56	0.94	0.52
Gaze direction bias	1.68	2.62	0.23
Gaze duration bias	0.27	1.50	0.58


Concerning the comparison between sham vs. actual feeding conditions, the Bayes factor of RT bias in 500 ms trials was 0.228, suggesting that the Bayes factors support the moderate evidence for null hypothesis (Schönbrodt and Wagenmakers, 2017).

### Experiment 2

#### Appetite Ratings

**Figure 2B** illustrates the mean standardized scores for subjective appetite in Experiment 2. In all appetite ratings, significant main effects of time and condition, and a significant interaction were observed (*p* < 0.001, **Table 1**).

In the sham feeding condition, significant simple main effects of time were observed on preoccupation with food [*F*(3,114) = 6.5, *p* < 0.001, η^2^ = 0.14] and desire to eat [*F*(3,114) = 2.9, *p* = 0.004, η^2^ = 0.07]. There was a marginal simple main effect on hunger [*F*(3,114) = 2.5, *p* = 0.059, η^2^ = 0.06]. *Post hoc* Ryan’s tests revealed significant decreases between T2 and T3 in preoccupation with food [*t*(114) = 3.8, *p* < 0.001, *r* = 0.34] and desire to eat [*t*(114) = 3.0, *p* = 0.004, *r* = 0.27] (**Table 2**).

In the actual feeding condition, a significant simple main effect of time was observed for hunger [*F*(3,114) = 82.0, *p* < 0.001, η^2^ = 0.68], fullness [*F*(3,114) = 110.0, *p* < 0.001, η^2^ = 0.74], preoccupation with food [*F*(3,114) = 79.5, *p* < 0.001, η^2^ = 0.68], and desire to eat [*F*(3,114) = 100.7, *p* < 0.001, η^2^ = 0.73]. *Post hoc* Ryan’s tests revealed significant decreases between T2 and T3 in hunger, preoccupation with food, and desire to eat, and a significant increase in fullness (*p* < 0.001 for all, **Table 2**).

The Bayes factors of appetite ratings for the comparisons between T2 vs. T3 were presented in **Table 3**. In the sham feeding condition, the Bayes factors of all appetite ratings support the alternative hypothesis; preoccupation with food and desire to eat support very strong evidence, hunger and fullness support moderate evidence for alternative hypothesis. In the actual feeding condition, the Bayes factors of all appetite ratings support extreme evidence for alternative hypothesis (Schönbrodt and Wagenmakers, 2017).

#### RT Bias Scores

**Figure 3B** and **Table 4** shows the RT bias scores in 2,000 ms trials obtained from the VPT in Experiment 2. No significant main effects of time [*F*(1,19) = 3.89, *p* = 0.06, η^2^ = 0.17] or condition [*F*(1,19) = 0.009, *p* = 0.92, η^2^ = 0.0005], nor any significant interaction between the two factors [*F*(1,19) = 0.81, *p* = 0.38, η^2^ = 0.04] were observed.

The Bayes factors of RT bias in 2,000 ms trials for the comparisons between before vs. after sham/actual feeding conditions were presented in **Table 5**. The Bayes factors of sham feeding and actual feeding conditions support anecdotal evidence for null hypothesis (Schönbrodt and Wagenmakers, 2017).

Regarding the comparison between sham vs. actual feeding conditions, the Bayes factor of RT bias in 2,000 ms trials was 0.333, suggesting that the Bayes factor supports moderate evidence for the null hypothesis (Schönbrodt and Wagenmakers, 2017).

#### Gaze Direction Bias Scores

**FIGURE 4:**
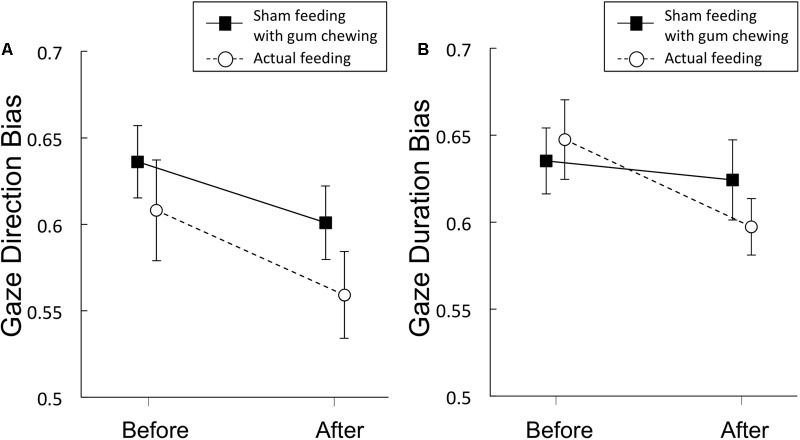
Gaze direction bias scores **(A)** and gaze duration bias scores **(B)** from Experiment 2. Solid lines represent sham feeding with gum-chewing sessions, and dashed lines represent actual feeding sessions. Error bars represent the standard error of the mean.

Gaze direction bias scores are presented in **Figure 4A** and **Table 4**. A significant main effect of time was observed [*F*(1,19) = 12.3, *p* = 0.002, η^2^ = 0.39], but no significant main effect of condition [*F*(1,19) = 2.77, *p* = 0.11, η^2^ = 0.13], nor any interaction of the two factors [*F*(1,19) = 0.27, *p* = 0.61, η^2^ = 0.014] emerged, indicating that sham feeding with gum-chewing reduced gaze direction bias as well as the actual feeding condition did.

The Bayes factors of gaze direction bias for the comparisons between before vs. after sham/actual feeding conditions were presented in **Table 5**. The Bayes factors of sham feeding and actual feeding conditions support anecdotal evidence for alternative hypothesis (Schönbrodt and Wagenmakers, 2017).

Regarding the comparison between sham vs. actual feeding conditions, the Bayes factor of gaze direction bias was 0.262, suggesting that the Bayes factor supports moderate evidence for the null hypothesis (Schönbrodt and Wagenmakers, 2017).

#### Gaze Duration Bias Scores

Gaze duration bias scores are presented in **Figure 4B** and **Table 4**. Marginally significant interaction were observed [*F*(1,19) = 3.81, *p* = 0.07, η^2^ = 0.17], although no significant main effects of time [*F*(1,19) = 2.77, *p* = 0.11, η^2^ = 0.13] or condition [*F*(1,19) = 0.231, *p* = 0.64, η^2^ = 0.01] were observed.

The Bayes factors of gaze duration bias for the comparisons between before vs. after sham/actual feeding conditions were presented in **Table 5**. The Bayes factor of sham feeding condition supports moderate evidence for null hypothesis and the Bayes factor of actual feeding condition supports anecdotal evidence for alternative hypothesis (Schönbrodt and Wagenmakers, 2017).

Regarding the comparison between sham vs. actual feeding conditions, the Bayes factor of gaze duration bias was 1.112, suggesting that the Bayes factor supports anecdotal evidence for the alternative hypothesis (Schönbrodt and Wagenmakers, 2017).

### Control Study

#### Appetite Ratings

**FIGURE 5:**
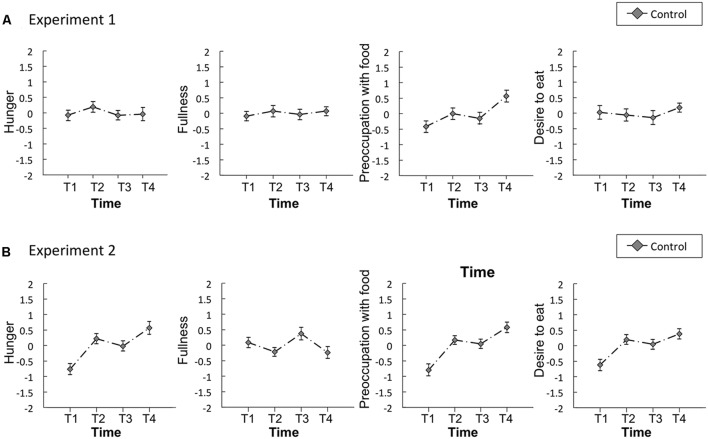
Time series of the standardized visual analog scales (VASs) for appetite ratings of hunger, fullness, preoccupation with food, and desire to eat in the control study for Experiments 1 **(A)** and 2 **(B)**. Error bars represent the standard error of the mean. T1, before the first visual probe tasks (VPT) or eye-tracking (ET); T2, before resting state; T3, after resting state; and T4, after the second VPT or ET.

**Figures 5A,B** illustrates the mean standardized scores of subjective appetite from the control study for Experiments 1 and 2, respectively. The Bayes factors of appetite ratings for the comparisons between T2 vs. T3 were presented in **Table 3**.

Regarding the control study for Experiment 1, significant main effects of time were observed for preoccupation with food [*F*(3,60) = 3.80, *p* = 0.01, η^2^ = 0.16]. A *post hoc* Ryan’s test revealed no significant decreases between T2 and T3 in all appetite ratings. However, there were significant differences between T1 and T4 in preoccupation with food [*t*(60) = 3.3, *p* = 0.002, *r* = 0.39] (Supplementary Table S4). The Bayes factors of all appetite ratings except for hunger support the moderate evidence for the null hypothesis and hunger supports anecdotal evidence for the null hypothesis (Schönbrodt and Wagenmakers, 2017).

Concerning the control study for Experiment 2, significant main effects of time were observed for hunger [*F*(3,57) = 7.38, *p* < 0.01, η^2^ = 0.28], preoccupation with food [*F*(3,57) = 9.54, *p* < 0.01, η^2^ = 0.33], and desire to eat [*F*(3,57) = 5.34, *p* < 0.01, η^2^ = 0.22]. A *post hoc* Ryan’s test revealed no significant decreases between T2 and T3 in all appetite ratings. However, there were significant differences between T1 and T2 [*t*(57) = 3.4, *p* = 0.001, *r* = 0.40], T1 and T3 [*t*(57) = 2.5, *p* = 0.014, *r* = 0.31], T1 and T4 [*t*(57) = 4.5, *p* < 0.001, *r* = 0.51] in hunger, T1 and T2 [*t*(57) = 3.7, *p* < 0.001, *r* = 0.43], T1 and T3 [*t*(57) = 3.2, *p* = 0.002, *r* = 0.38], T1 and T4 [*t*(57) = 5.2, *p* < 0.001, *r* = 0.56] in preoccupation with food, T1 and T2 [*t*(57) = 3.1, *p* = 0.003, *r* = 0.37], T1 and T3 [*t*(57) = 2.5, *p* = 0.015, *r* = 0.31], T1 and T4 [*t*(57) = 3.7, *p* < 0.001, *r* = 0.44] in desire to eat (Supplementary Table S4). The Bayes factors of all appetite ratings except for fullness support the null hypothesis; preoccupation with food and desire to eat support moderate evidence and hunger support anecdotal evidence for null hypothesis. Only fullness supports anecdotal evidence for the alternative hypothesis (Schönbrodt and Wagenmakers, 2017).

#### RT Bias Scores

**FIGURE 6:**
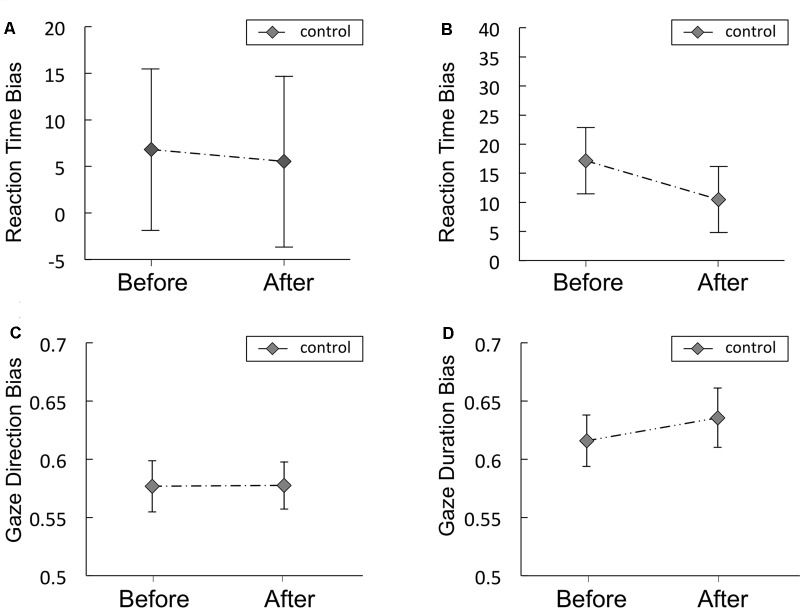
Reaction time bias scores (in ms) obtained from the visual probe tasks in the control study for Experiments 1 **(A)** and 2 **(B)**, Gaze direction bias scores **(C)** and gaze duration bias scores **(D)** from Experiment 2. The food and non-food images were presented for 500 ms **(A)** or 2,000 ms **(B)**. Error bars represent the standard error of the mean.

**Figures 6A,B** shows the RT bias scores obtained from the VPT in the control study for Experiments 1 and 2, respectively. Mean scores are shown in Supplementary Table S5. No significant difference was observed between before and after in the RT bias scores in the control study for Experiments 1 or 2. The Bayes factors of RT bias for the comparisons between before vs. after resting were presented in **Table 5**. The Bayes factor of RT bias in 500 ms trials supports moderate evidence for null hypothesis and the Bayes factor of RT bias in 2,000 ms trials supports anecdotal evidence for null hypothesis (Schönbrodt and Wagenmakers, 2017).

#### Gaze Direction and Duration Bias Scores

Gaze direction and duration bias scores in the control study are presented in **Figures 6C,D**, respectively. Mean scores are shown in Supplementary Table S5. No significant difference was observed between before and after in gaze direction or duration bias scores. The Bayes factors of gaze direction and duration bias for the comparisons between before vs. after resting condition were presented in **Table 5**. The Bayes factor of gaze direction bias supports moderate evidence for null hypothesis and the Bayes factor of gaze duration bias supports anecdotal evidence for null hypothesis (Schönbrodt and Wagenmakers, 2017).

## Discussion

Using VPT and ET, this study investigated that the effects of chewing stimulation on appetite from the perspective of the reward pathway, and compared these effects in a sham feeding (gum-chewing) condition with those in an actual feeding condition.

Subjective appetite ratings in the actual feeding condition showed that hunger, preoccupation with food, and desire to eat were reduced significantly, and fullness increased significantly after feeding (Experiments 1 and 2). Similarly, after sham feeding with gum-chewing, ratings of hunger (Experiment 1), preoccupation with food (Experiments 1 and 2), and desire to eat (Experiment 2) decreased significantly, and fullness increased significantly (Experiment 1). Additionally, hunger ratings in Experiment 2 and desire to eat in Experiment 1 showed marginally significant increases. Thus, a reduction in subjective appetite after chewing calorie free gum, although not as much as in the actual feeding session, suggests that chewing stimulation itself might reduce appetite. These results were supportive of findings from previous studies that chewing sugarless gum increased satiety on VAS (Xu et al., 2015).

In a systematic review of the effects of chewing on appetite using the meta-analysis approach, 15 papers were extracted, six of which were involved in the effect of gum chewing on appetite. Three papers’ results showed significant changes in subjective appetite, but two papers showed no significant changes (Miquel-Kergoat et al., 2015). The possible causes of these inconsistent results are those of chewing being subjective to thresholds (Miquel-Kergoat et al., 2015) in its duration (Shikany et al., 2012), its timing (Julis and Mattes, 2007), number of chews (Mattes and Considine, 2013; Zhu et al., 2013). In this study, even though there was no difference in the sham feeding condition between Experiments 1 and 2, such as duration of chewing, timing, and numbers of chews, different appetite ratings resulted. Perhaps, therefore, individuals may respond differently to the chewing stimulation’s effect on appetite.

Additionally, in Experiments 1 and 2, significantly increased appetite ratings from T1 to T2 were observed only in preoccupation with food, while other appetite ratings were not altered significantly. It is assumed that visual food stimuli during the tasks made participants more preoccupied with food, and it can be said that appetite ratings generally were not increased by visual food stimuli. However, under control conditions, the ratings of not only preoccupation with food, but also hunger and desire to eat, significantly increased from T1 to T2.

Moreover, appetite ratings of hunger, preoccupation with food, and desire to eat all tended to decrease after sham feeding with gum-chewing in both experiments. A previous study had suggested that appetite-related drives to eat – for example, hunger, preoccupation with food, and the desire to eat – were influenced more by physiological, environmental, social, and behavioral factors than by the diet’s energy content and macronutrient composition (Leidy et al., 2011). In addition, feelings of hunger might reflect the desire to eat (Harris et al., 2008), which might then evolve into craving (Pelchat et al., 2004). Conversely, satiety appeared to be less driven by those factors (Leidy et al., 2011). Indeed, it has been reported that increasing gastric distention and the infusion of gastric hormones (e.g., cholecystokinin) increases fullness ratings but does not reliably alter hunger ratings (Melton et al., 1992; Harris et al., 2008). Therefore, ratings of hunger, preoccupation with food, and desire to eat seem to have similar appetite properties that reflect more feelings of craving, whereas fullness may be a different type of indicator. There were no significant changes between T2 and T3 in any of the control conditions, suggesting that chewing stimulation by itself might reduce feelings of craving.

The Bayesian analyses show clear evidence for a difference between T2 vs. T3 appetite ratings for the sham and actual feeding condition, albeit substantially stronger evidence for a difference in the actual condition. Furthermore, there is evidence for no difference across the majority of appetite ratings in the control study. The pattern observed for the sham and actual feeding conditions also replicates across Experiments 1 and 2. Combined, it can be interpreted that the effects of chewing stimulation and actual feeding on appetite ratings are clearly strong effects.

The results of VPT in Experiment 1 demonstrate that RT bias scores are reduced significantly after sham feeding with gum-chewing and after actual feeding; i.e., not only ingesting a meal, but also chewing tasteless, odorless, calorie-free gum reduced attentional bias to food. However, the results of the VPT in Experiment 2 showed no significant main effects and no interaction. It could therefore follow that the use of longer presentation durations, such as 2,000 ms, allows multiple shifts in attention between two adjacent images (Nijs et al., 2010).

It has been suggested that initial attentional orientation could be evaluated with a presentation time of 200 ms or less (Field and Cox, 2008). Whereas, Armstrong and Olatunji (2012) suggested that fixation duration in the first 500 ms provided a valid measure of initial orienting. Thus, interpretation regarding VPT presentation time is inconsistent, and mentioning initial or maintained attention is assumed to be difficult for the present study’s VPT results for image presentation time. In the present study, at least, the result of VPT as a preliminary investigation showed reduced attentional bias after sham feeding with gum-chewing.

The results of ET show a significant reduction in the gaze direction bias score, which is considered to reflect initial attentional orientation (Castellanos et al., 2009), after sham feeding and actual feeding. In contrast, there were no significant main effects in the gaze duration bias score, which is considered to be an index of maintained attention (Castellanos et al., 2009). Therefore, the present study’s results suggest that chewing stimulation, similar to actual feeding, reduces initial attentional orientation to food. Initial attention involves an involuntary, automatic, passive, bottom-up attentional process (Nijs, 2010), suggesting that chewing stimulation – even when it lacks taste, odor, or ingestion – might affect involuntary attention. In addition, it has also been said that salience generally captures initial attention (Sawaki and Luck, 2010). Thus, chewing stimulation may suppress the salience of food stimuli and in this way, potentially prevent cravings for food. Bazzaz et al. (2017) suggested that reducing attentional bias to food through food attention control training might positively impact eating behavior. In our study, attentional bias to food did not completely disappear but significantly decreased after sham feeding and actual feeding, suggesting the possibility of chewing stimulation’s influence on impulsive eating behavior.

The specific disinhibited eating pattern in binge eating disorder (BED) patients has promoted the assumption that BED might represent a phenotype within the obesity spectrum that is characterized by increased impulsivity (Giel et al., 2017). Food-related reward sensitivity and rash-spontaneous behavior, as the two components of impulsivity, are increased in BED (Schag et al., 2013). Moreover, it has been reported that an increased initial attention toward food is related to greater BED symptomatology (Sperling et al., 2017). Thus, reduced initial attention to food stimuli by prompting masticatory activity might be effective against BED.

The hitherto described mechanism of chewing stimulation’s effect on appetite has typically involved two aspects. First, chewing stimulation is a crucial factor in cephalic phase responses, and it may increase the gastric or hormonal release of substances related to appetite, such as pancreatic polypeptide and cholecystokinin (Teff, 2000; Zhu et al., 2013; Veedfald et al., 2016). Second, animal studies have shown that mastication-induced activation of histamine neurons suppresses physical food intake through the H-1 receptor in the hypothalamic paraventricular nucleus and the ventromedial hypothalamus, which together are known as the satiety center (Sakata et al., 2003).

Furthermore, this study led to a new insight into the mechanism of reducing appetite by chewing stimulation: chewing stimulation reduces attentional bias to food, and might affect the reward pathway because the attentional bias to food stimuli could be a behavioral output of the individuals’ reward circuits (Jonker et al., 2016).

No reports have specifically investigated the association between chewing stimulation and the reward circuits; however, some reports support this hypothesis. One previous study demonstrated that the dopamine turnover in the frontal cortex was elevated in mice that were fed a powdered diet (less chewing) compared with those fed a standard diet (control) (Niijima-Yaoita et al., 2013). Another study revealed that dopamine activity in rats was suppressed; that is, the rats made less effort to obtain food, and food intake was therefore reduced (Wise and Rompre, 1989). Considering these studies, it can be assumed that chewing stimulation might inhibit the secretion of dopamine, which could contribute to the suppression of appetite.

It is notable, however, that the Bayes factors for the before vs. after RT bias and gaze direction bias differences do not provide convincing evidence for either the alternative or null hypotheses in both the sham and actual feeding conditions. There might be several possible reasons why not so strong evidence was provided in these conditions in the present study.

First, participants in the actual feeding condition completed the VPT or were subjected to ET immediately after food ingestion. It is therefore possible that postprandial hormones had not yet sent satiety signals to the brain, even though participants did report a significant decrease in appetite. In future studies, including a time delay seems advisable, as exemplified by Castellanos et al. (2009).

Second, the duration of gum chewing in this study was much shorter than those in previous studies reporting chewing’s effect on appetite (Miquel-Kergoat et al., 2015). With regard to the actual feeding session, the time required to “feeling satiated” by an energy supplement ingestion was preliminarily verified to take approximately 5 min. Accordingly, we set gum-chewing time to 5 min to match the actual feeding time as much as possible. However, Miquel-Kergoat et al. (2015) reported that the chewing effect was obtained after more than 10 min chewing time. Thus, in further research, we would like to consider a longer chewing period. In addition to this, we would like to consider long-term effects of gum chewing on appetite.

Thirdly, we conducted control study with different participants in Experiments 1 and 2, so that we could not compare among the effects of chewing, actual feeding, and control conditions in within-subject design due to limitations of our present study. Therefore, further investigations would be needed to give light on the effect of mastication on satiety, which might be beneficial in preventing impulsive eating especially in people with obesity.

## Conclusion

We have demonstrated that chewing stimulation can reduce subjective appetite ratings that reflect craving. Moreover, chewing alone, as with feeding, results in reduced attentional bias to food, which has been suggested to be a behavioral output of the individual’s reward system, and particularly the initial attentional orientation to food, although the Bayes factors do not provide strong evidence for the alternative hypotheses in both the sham and actual feeding conditions. These findings possibly suggest that appetite reduction by chewing stimulation, even in the absence of taste, odor, and ingestion, may affect reward circuits and help prevent impulsive eating. We therefore propose that interventions directed toward stimulation of chewing activity could become a valuable adjunct tool for controlling the drive to eat.

## Author Contributions

AI has conceived the study. All authors made a substantial and intellectual contribution to the work, participated in writing the manuscript and approved it for publication. AI, JM, and NU contributed to the design, data collection, analysis, and writing. MT and KM contributed to the design and writing.

## Conflict of Interest Statement

The authors declare that the research was conducted in the absence of any commercial or financial relationships that could be construed as a potential conflict of interest. The reviewer MB and handling Editor declared their shared affiliation.

## Abbreviations

ET: eye-tracking
RT: reaction time
VPT: visual probe task
