# Supplementary material for: Chewing Stimulation Reduces Appetite Ratings and Attentional Bias toward Visual Food Stimuli in Healthy-Weight Individuals
Source: Front Psychol. 2018 Feb 8;9:99. doi: 10.3389/fpsyg.2018.00099 (PMC5809478; doi:10.3389/fpsyg.2018.00099)
Supplement: Supplementary file 1 [file Data_Sheet_1.pdf]

Chewing stimulation reduces appetite ratings and appetite ratings and attentional bias toward visual food stimuli in healthy-weight individuals

Authors: Akitsu Ikeda[1], Jun J. Miyamoto[1*], Nobuo Usui[2], Masato Taira[2], Keiji Moriyama[1]

[1] Maxillofacial Orthognathics, Division of Maxillofacial and Neck Reconstruction, Graduate School of Medical and Dental Sciences, Tokyo Medical and Dental University (TMDU), Tokyo, Japan

[2] Cognitive Neurobiology, Division of Maxillofacial and Neck Reconstruction, Graduate School of Medical and Dental Sciences, Tokyo Medical and Dental University (TMDU), Tokyo, Japan

***Correspondence:**

Jun J. Miyamoto

mjmort@tmd.ac.jp

Keywords: chewing, attentional bias, visual probe task, eye-tracking, reward circuit, appetite

Supplementary Material

This study was conducted at the Tokyo Medical and Dental University in Japan. Through campus flyers and e-mails, the students of the university were informed about the present study and asked to contact us (via email) if they were interested in participating. A computer-generated list of random numbers was used to assign the participants to one of the two order conditions: sham feeding with gum-chewing first, or actual feeding first. An investigator (AI) generated the allocation sequence, and co-author (JJM), who was not in contact with the participants during the trial period, enrolled and assigned the participants to the conditions. Within a sixty-day period, participants attended two sessions of experiments separated by a two-week interval between the sessions.

Each image used in the visual probe task (VPT) and the eye-tracking (ET) procedure was suspended at a visual angle of 8.8° × 12.5°. The distance between centers of paired images was 16.5°. E-prime 2.0 (Psychology Software Tools, Inc. Pittsburgh, PA, USA) was used for stimulus presentation and for recording behavioral responses. During VPT, participants were positioned in front of a monitor with their chins securely placed on a chin rest to maintain the distance of 71.5 cm from the monitor. Participants began with 16 practice trials that did not include images used in the main study.

To examine relationships between appetite ratings (the difference between T2 and T3 in ratings of hunger, fullness, preoccupation with food, and desire to eat) and attentional bias (the difference between before and after in RT bias, gaze direction bias, and gaze duration bias), Pearson correlations were computed (Supplementary Table S3). On the whole in this study, correlations between subjective appetite ratings and attentional bias were shown to be very weak. Only fullness showed a relatively strong and significant negative correlation with gaze duration bias in sham feeding condition. Given this finding, it is assumed that the degree of change in subjective appetite ratings and the degree of change in attentional bias score may not always match. Thus, further investigations would be required to discuss the relevance between subjective appetite and attentional bias.

In Experiment 2, 8 participants were excluded due to a lack of sufficient ET data. We removed these data from all the tasks to match the sample numbers among participants. When the data of the 8 excluded participants were included in the visual analog scales (VASs) and VPTs, the results were as follows.

VAS. Figure S2 illustrates the mean standardized scores for subjective appetite in Experiment 2. In all appetite ratings, the significant main effects of time and condition and a significant interaction were observed ($p < 0.001$). In the sham feeding condition, a simple significant main effect of time was observed for hunger [$F(3, 162) = 3.3$, $p = 0.023$, $\eta^2 = 0.06$], fullness [$F(3, 162) = 3.6$, $p = 0.015$, $\eta^2 = 0.06$], and preoccupation with food [$F(3, 162) = 10.2$, $p < 0.001$, $\eta^2 = 0.16$]. Post hoc Ryan's tests revealed a significant decrease between T2 and T3 in fullness [$t(162) = 3.0$, $p = 0.003$, $r = 0.23$] and in preoccupation with food [$t(162) = 3.9$, $p < 0.001$, $r = 0.29$]. In the actual feeding condition, a significant simple main effect of time was observed for hunger [$F(3, 162) = 63.4$, $p < 0.001$, $\eta^2 = 0.54$], fullness [$F(3, 162) = 116.8$, $p < 0.001$, $\eta^2 = 0.68$], preoccupation with food [$F(3, 162) = 111.3$, $p < 0.001$, $\eta^2 = 0.67$], and desire to eat [$F(3, 162) = 121.2$, $p < 0.001$, $\eta^2 = 0.69$]. Post hoc Ryan's tests revealed a significant decrease between T2 and T3 in hunger, preoccupation with food, and desire to eat, and a significant increase in fullness ($p < 0.001$ for all).

RT bias scores. No significant main effects of time or condition, nor any significant interaction was observed.

Table S1. Mean proportion of acquired samples of eye-tracking data

Condition	Sham feeding		Actual feeding		Control	
Time	Before	After	Before	After	Before	After
Mean	82.35	83.55	82.70	84.55	76.25	76.80
SD	8.87	7.55	9.13	8.46	13.22	11.38

Table S2. Mean ratings of valence, arousal, and appetite for each stimulus set

	Food						Nonfood				Filler (Nonfood)			
	Valence		Arousal		Appetite		Valence		Arousal		Valence		Arousal	
	Mean	SD	Mean	SD	Mean	SD	Mean	SD	Mean	SD	Mean	SD	Mean	SD
1	7.20	0.31	6.29	6.26	4.13	4.15	5.10	0.27	3.19	0.62	6.45	0.85	4.14	1.19
2	7.18	0.25	6.38	0.34	4.19	0.15	4.95	0.44	3.17	0.78	6.65	0.85	4.30	1.06
3	7.12	0.26	6.20	0.54	4.14	0.15	4.92	0.25	2.93	0.59	6.71	0.81	4.39	1.08
4	7.20	0.30	6.29	0.38	4.15	0.16	5.05	0.50	2.89	0.63	6.74	0.86	4.29	0.90

The affective ratings of nonfood images were obtained from the normative ratings of the International Affective Picture System images.

Table S3. Pearson's correlation coefficient between subjective appetite and attentional bias scores

	Subjective appetite ratings (T3-T2)	Attentional bias (T3-T2)							
		RTbias (500 ms)		RTbias (2000 ms)		Direction bias		Duration bias	
		r	*p*	*r*	*p*	*r*	*p*	*r*	*p*
Sham feeding session	Hunger	-0.09	0.689	0.31	0.190	-0.20	0.393	0.31	0.191
	Fullness	-0.01	0.961	0.10	0.689	-0.02	0.946	-0.54	0.015
	Preoccupation with food	-0.19	0.419	-0.22	0.362	-0.27	0.258	0.12	0.619
	desire to eat	-0.17	0.469	-0.13	0.576	-0.26	0.271	0.31	0.184
Feeding session	Hunger	-0.15	0.530	0.09	0.699	0.36	0.118	0.21	0.384
	Fullness	0.07	0.758	-0.256	0.276	-0.29	0.212	-0.02	0.929
	Preoccupation with food	-0.25	0.266	0.31	0.184	0.41	0.075	0.19	0.417
	desire to eat	-0.40	0.078	-0.07	0.757	0.15	0.535	-0.11	0.634

Table S4. Mean standardized appetite ratings (standard errors) as assessed at four time points during the control experiment

			Time Points				
			T1	T2	T3	T4	Significant difference
			Mean ± SE	Mean ± SE	Mean ± SE	Mean ± SE	
Experiment 1	Hunger	Control	-0.07 ± 0.16	0.20 ± 0.16	-0.08 ± 0.14	-0.04 ± 0.20	
	Fullness	Control	-0.10 ± 0.15	0.07 ± 0.18	-0.04 ± 0.17	0.07 ± 0.15	
	Preoccupation with food	Control	-0.42 ± 0.17	-0.001 ± 0.19	-0.15 ± 0.19	0.57 ± 0.19	c
	Desire to eat	Control	0.02 ± 0.21	-0.06 ± 0.19	-0.15 ± 0.21	0.18 ± 0.14	
Experiment 2	Hunger	Control	-0.76 ± 0.18	0.22 ± 0.16	-0.02 ± 0.16	0.57 ± 0.21	a,b,c
	Fullness	Control	0.08 ± 0.17	-0.22 ± 0.14	0.37 ± 0.20	-0.24 ± 0.19	
	Preoccupation with food	Control	-0.80 ± 0.19	0.17 ± 0.14	0.05 ± 0.15	0.58 ± 0.17	a,b,c
	Desire to eat	Control	-0.62 ± 0.18	0.20 ± 0.15	0.04 ± 0.16	0.38 ± 0.16	a,b,c

significant difference, a: T1vsT2, b:T1vsT3, c: T1vsT4, d:T2vsT3, e:T2vsT4, f:T3vsT4

Table S5. Mean scores for attention-related measures before and after resting state in the control session

	Control study	
	Before	After
	Mean ± SE	Mean ± SE
**Experiment 1 (control)**		
(Stimulus duration = 500ms)		
RT food	315.07 ± 10.04	308.46 ± 8.09
RT nonfood	321.89 ± 15.26	313.99 ± 12.83
RT bias	6.83 ± 8.65	5.53 ± 9.14
**Experiment 2 (control)**		
(Stimulus duration = 2000ms)		
RT food	373.51 ± 17.73	365.22 ± 14.32
RT nonfood	390.65 ± 19.70	375.71 ± 17.06
RT bias	17.14 ± 5.68	10.49 ± 5.63
Gaze direction food	29.60 ± 2.36	30.10 ± 2.44
Gaze direction nonfood	20.85 ± 1.61	21.35 ± 1.61
Gaze direction bias	0.58 ± 0.02	0.58 ± 0.02
Gaze duration food	642.70 ± 67.33	714.68 ± 74.81
Gaze duration nonfood	396.89 ± 40.83	382.69 ± 39.45
Gaze duration bias	0.62 ± 0.02	0.64 ± 0.03

Figure S1. Study protocol.

VAS, visual analog scale; VPT, visual probe task; ET, eye-tracking

Figure S2. Time series of the standardized visual analog scales (VASs) of appetite ratings of hunger (A), fullness (B), preoccupation with food (C), and desire to eat (D), for the 28 participants in Experiment 2. Solid lines represent sham feeding with gum-chewing sessions, and dashed lines represent actual feeding sessions. Error bars denote the standard error of the mean. T1, before the first eye-tracking (ET); T2, before sham feeding or actual feeding; T3, after sham feeding or actual feeding; and T4, after the second ET.
