# Supplementary figures and images for: Chewing Stimulation Reduces Appetite Ratings and Attentional Bias toward Visual Food Stimuli in Healthy-Weight Individuals
Source: Front Psychol. 2018 Feb 8;9:99. doi: 10.3389/fpsyg.2018.00099 (PMC5809478; doi:10.3389/fpsyg.2018.00099)

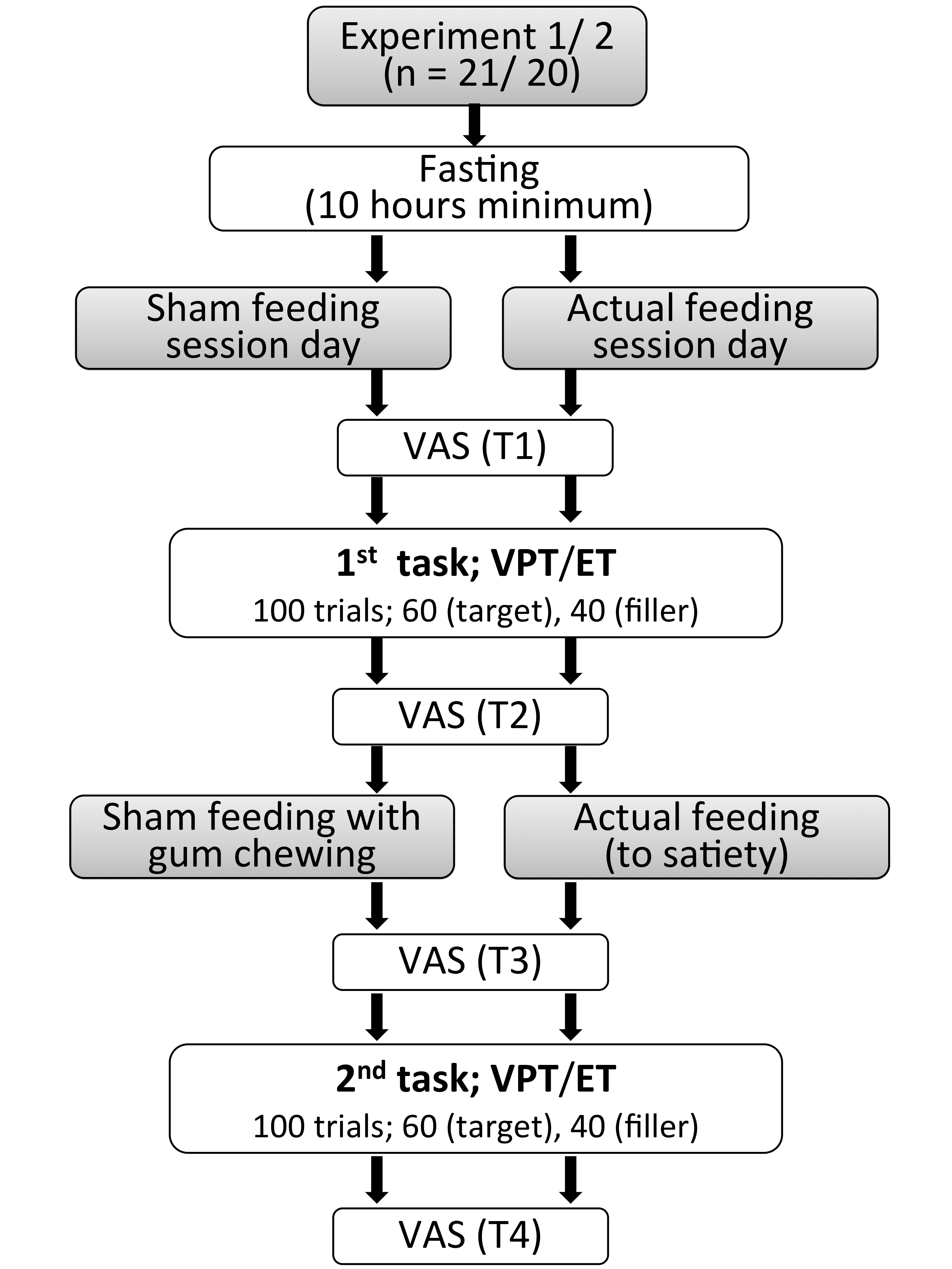

Supplement: Supplementary file 2 [file Image_1.JPEG]

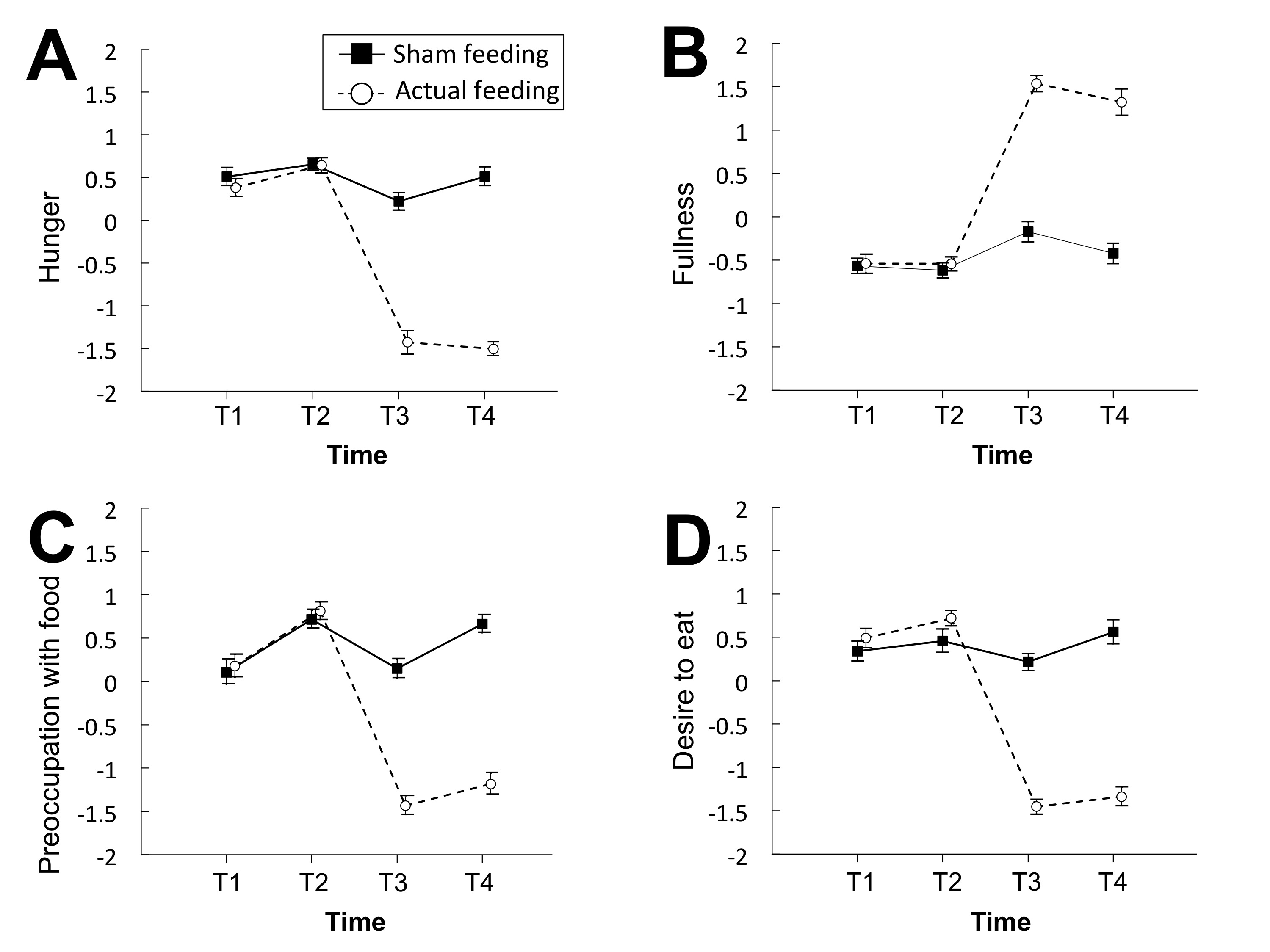

Supplement: Supplementary file 3 [file Image_2.JPEG]
